# FT-IR Method Limitations for β-Glucan Analysis

**DOI:** 10.3390/molecules27144616

**Published:** 2022-07-20

**Authors:** Ruslan Bikmurzin, Rimantė Bandzevičiūtė, Arūnas Maršalka, Andrius Maneikis, Lilija Kalėdienė

**Affiliations:** 1 Department of Microbiology and Biotechnology, Institute of Biosciences, Life Sciences Center, Vilnius University, Sauletekio av. 7, LT-10257 Vilnius, Lithuania; lilija.kalediene@gf.vu.lt; 2 Department of Medical Technology and Dietethics, Faculty of Health Care, Vilnius University of Applied Sciences, Didlaukio str. 45, LT-08303 Vilnius, Lithuania; 3 Institute of Chemical Physics, Faculty of Physics, Vilnius University, Saulėtekio av. 3, LT-10257 Vilnius, Lithuania; rimante.bandzeviciute@ff.vu.lt (R.B.); arunas.marsalka@ff.vu.lt (A.M.); 4 Department of Computer Science and Communications Technologies, Vilnius Gediminas Technical University, Saulėtekio av. 11, LT-10221 Vilnius, Lithuania; andrius.maneikis@vgtu.lt

**Keywords:** β-glucans, FT-IR, solid-state, NMR, β-1,3/1,6-linked glucans, yeast glucans

## Abstract

β-glucans are known as biological response modifiers. However, different sources can result in structural differences and as a result differences in their biological activity. The hot water extraction method allows to obtain, high molecular weight β-glucans without altering their structure by using strong chemicals, such as alkalis or acids. Analysis of β-glucans by FT-IR and NMR spectroscopy in solid state is superior to analysis in solution as it allows researchers to study the preserved structure of the extracted polysaccharides. FT-IR spectroscopy was used in this study to make side-by-side comparison analysis of hot water extracted β-glucans from different yeast sources. NMR spectroscopy was used to confirm findings made by FT-IR spectroscopy. Extracted β-glucans exhibit characteristic structure of β-1,3/1,6-linked glucans with noticeable levels of proteins, possibly in a form of oligopeptides, chitin and other impurities. β-glucans obtained from *C. guilliermondii*, *P. pastoris* and *S. pastorianus* exhibited higher protein content. Differences in mannan, chitin and α-glucan content were also observed; however, the species-specific structure of obtained β-glucans could not be confirmed without additional studies. Structural analysis of high molecular weight β-glucans in solid state by FT-IR spectroscopy is difficult or limited due to band intensity changes and overlapping originating from different molecules.

## 1. Introduction

β-glucans are a heterogeneous group of biologically active polysaccharide polymers whose main characteristic is glucose monomers linked together by β-glycosidic bonds. β-glucans are widely spread among different microorganisms, fungi and plants [1]. However, they are mainly extracted from mushrooms, yeasts, seaweed and crops, such as oats and barley [2,3]. β-glucan sources result in their structural differences; for example, yeast and fungi β-glucans mainly consist of backbone glucose monomers linked by β-1,3- and side branches attached by β-1,6-glycosydic linkage [4]. Glucan from algae is mostly a linear β-1,3-glucan, while from oat and barley, it is a linear β-1,4-glucan [5]. β-glucans are known for their immunomodulatory functions. Their position in the cell wall of pathogens make them a target for immune response. Because of the effect of β-glucans on the immune system, they are called biological response modifiers (BRM) [3]. As immunomodulators, they enhance both innate and acquired host immune functions through binding to specific receptors on macrophages, natural killer (NK) and neutrophils and activation of different cellular pathways [6,7]. β-glucans are used as a dietary supplement and as part of anticancer or anti-inflammatory therapy [8,9]. Their wound healing properties are also studied, and they are used in cosmetic products due to antioxidative and immunological properties [4,10]. These biologically active compounds have a great potential for medical application, with many completed or ongoing clinical trials [4,7,8,10,11,12]. For example, β-glucans can be used as potent carrying systems for drug delivery or as matrix for biomaterials [13].

In yeasts, glucans are a major component of the cell wall and can comprise up to 60% of its mass, which in turn accounts for up to 30% of the cell dry mass [14,15,16,17]. The β-1,3-glucan carcass makes up to 85% of yeast glucans and another part is made from short-chained branches of β-1,6-glucan molecules. Other constituents of the cell wall are mannans accounting for up to 30% of its mass, chitin 1–2%, proteins up to 30% and lipids around 9%. Mannans, chitin and proteins attach to glucans through branched β-1,6-glucan molecules [14,18,19,20,21,22,23,24]. Due to the function of cell walls, molecules within are highly organized and linked together, which makes the isolation of β-glucan difficult, time and energy consuming multistep process [25]. The combination of physical, chemical and enzymatic methods is used for glucan isolation. Cell lysis and cell wall breaking is an essential step in glucan isolation. Different isolation methods affect the mass and branching of the β-glucans, which can lead to a wide range of molecular mass distribution, and level of branching, yield and purity of the final product can lead to structural differences of extracted β-glucans and, as a result, differences in their biological activity [1,16,23,25,26,27,28].

Insolubility of some β-glucans and chitin impedes their analysis in solution. Therefore, possessing difficulties in the application of usual enzymatic, chemolytic and chromatographic methods for the determination of molecular weight, composition and structure. In order to estimate molecular weight (MW), dissolution is desired; however, presence of aggregates in solution precludes light scattering and overestimates MW [29]. Another problem is associated with β-glucan content analysis. For example, neither phenol-sulphuric nor the DNS method can distinguish between the type of sugar present in the sample [30]. Other techniques, such as gas and liquid chromatography and mass spectrometry, are great for component determination. However, due to the necessity to hydrolyze the samples, residual proteins and lipids could interfere with the hydrolytic action of enzymes applied in the enzymatic β-glucans analysis, causing steric hindrance and, as a result, inefficient hydrolysis of the glycosidic bond. Thus, neither sonication nor chemical or enzymatic hydrolyzation guarantee adequate recovery of β-glucan content [19,31,32]. Hydrolysis of β-glucans does not interfere with accurate structural analysis. To circumvent this problem, analytical techniques, such as Fourier transform infrared (FT-IR) and nuclear magnetic resonance (NMR) spectroscopy are used. However, β-glucan analysis in solution requires its degradation, which may affect the results. Fortunately, both FT-IR and NMR as well as Raman spectroscopy and X-ray crystallography allow the use of solid samples [28,30,33]. Together with hot water extraction, this allows the analysis of the preserved native structure of the β-glucans [24,33]. FT-IR spectroscopy is a powerful technique which has been used to analyze glucans from yeasts and is suitable to analyze the position and anomeric configuration of glycosidic linkages in high molecular weight glucans isolated from raw materials, thus allowing researchers to evaluate structural differences of these molecules [31,34]. In this paper, FT-IR spectroscopy was used as the main method and solid-state NMR spectroscopy was used as the accompanying method to analyze β-glucan extracts from six different yeast strains: *Saccharomyces cerevisiae*, *Saccharomyces pastorianus*, *Candida guilliermondii*, *Candida lusitaniae*, *Candida parapsilosis* and *Pichia pastoris*.

Lack of information and side-by-side comparison of high molecular weight β-glucan analysis in solid state led us to this research. The aim of the present study was to evaluate structural differences of hot water extracted high molecular weight β-glucan fractions from different yeasts by using FT-IR spectroscopy.

## 2. Results

### 2.1. Polysaccharide Extraction

High molecular weight (HMW) β-glucan extracts were obtained by the hot water extraction method from six different yeast strains: *S. cerevisiae*, *S. pastorianus*, *C. guilliermondii*, *C. lusitaniae*, *C. parapsilosis* and *P. pastoris*. β-glucans comprise 40–60% of the *S. cerevisiae* cell wall dry mass, which in turn comprises 10–30% of the dry mass of yeast cells [19,21,24]. Other constituents, such as mannoproteins and chitin, comprise 30–50% and 1% of the cell walls’ dry mass, respectively. Chitin binding with β-1,3-glucan as well as high degree of polymerization of β-1,3-glucan make it insoluble [21,24].

The β-glucan amount in the cell wall is species-related and depends on various environmental factors. That is why in order to yield comparable data, all yeasts were grown in the same media under same conditions. In this study, the highest yield of HMW β-glucan was obtained from *S. pastorianus*, which made around 9% of the initial yeast biomass and around 58% of the cell wall mass (Table 1). The lowest β-glucan yield was from *P. pastoris*—4.99% of the initial yeast biomass. However, the lowest content of hot water-extracted β-glucans relative to the cell wall mass was from *C. lusitaniae* and *P. pastoris*, where glucan made around 37% and 38% of the cell wall mass, respectively. Overall, a higher yield of HMW β-glucan relative to initial biomass as well as to the mass of the cell wall was in *Saccharomyces* yeasts.

### 2.2. Scanning Electron Microscopy

**Figure 1:**
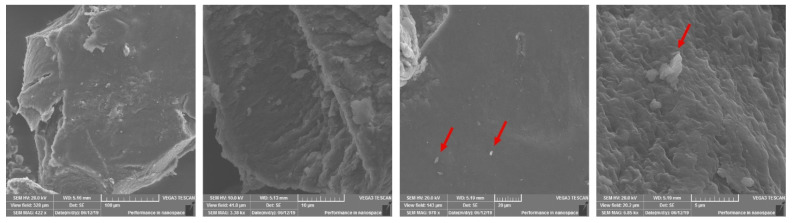
Different magnification SEM images of *S. cerevisiae* air-dried glucan particles after hot water extraction. Red arrows point to artefacts.

SEM was used to confirm structural characteristics of the glucan extracts. Air-dried insoluble glucan fractions suspended in distilled water resulted in a thin-layer, mechanically stable uniform structure, which makes it extremely difficult to grind into a fine powder. Air-dried glucans were grinded, obtaining β-glucan powder and particles of different sizes and were subjected to SEM analysis. After air-drying, β-glucan extracts were able to retain electric charge on their surface, which distorted the SEM images; therefore, particles were covered by a layer of gold particles. These particles were of irregular shape and sharp edges, with a significant degree of aggregation. They formed a flat, porous surface, which seems to be a highly organized β-glucan polymer structure. The surface had debris after grinding but also there were artefacts, which were visually connected to the surface layer of the particles (Figure 1). We guess that it could be remains of denaturated proteins bound to β-glucans. The cross-section reveals the inner multilayer or sheet-like structure of β-glucan particles. Drying methods might affect the structure of the β-glucan; however, obtained data is in agreement with similar studies [25].

### 2.3. Fourier-Transform Infrared Spectroscopy

**Figure 2:**
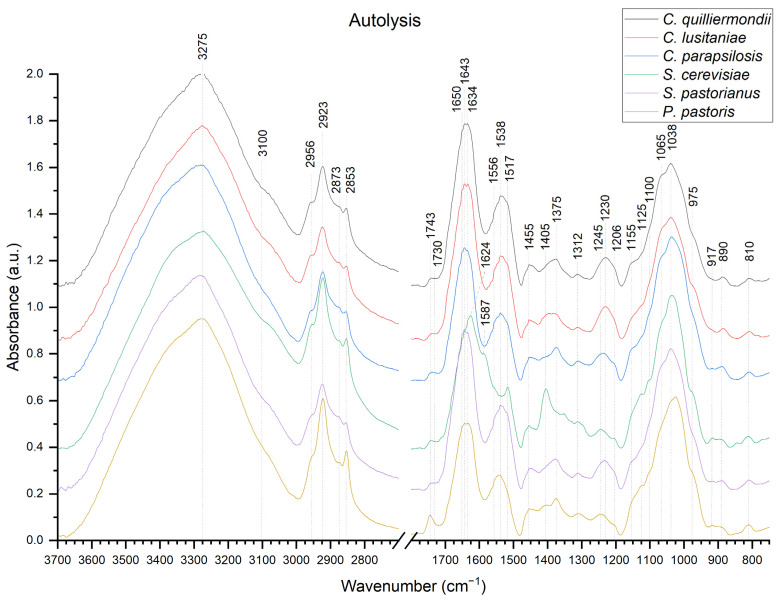
ATR IR absorption spectra of autolyzed cells of different yeast strains: (from top to bottom) *C. guilliermondii*, *C*. *lusitaniae*, *C*. *parapsilosis*, *P. pastoris*, *S. cerevisiae* and *S*. *pastorianus*.

**Figure 3:**
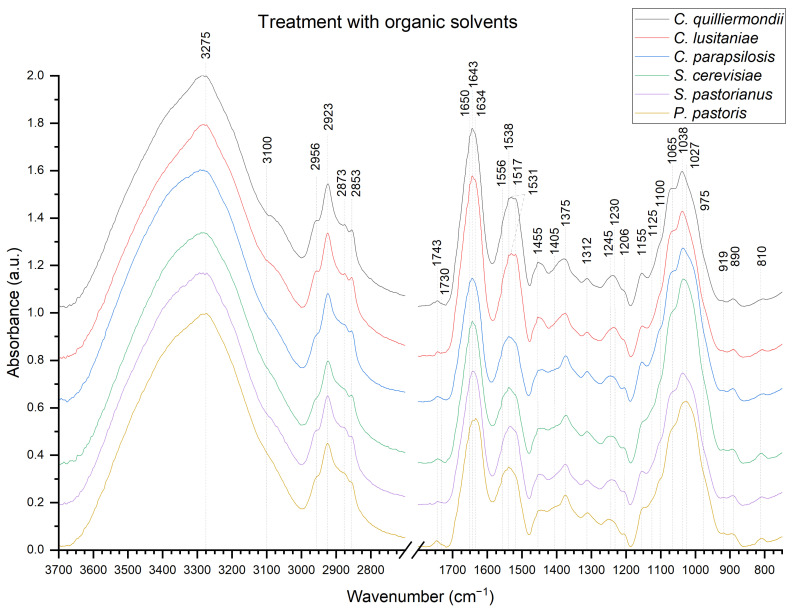
ATR IR absorption spectra of hot water-extracted β-glucans treated with organic solvent (EtOH) from different yeast strains: (from top to bottom) *C. guilliermondii*, *C*. *lusitaniae*, *C*. *parapsilosis*, *P. pastoris*, *S. cerevisiae* and *S*. *pastorianus*.

In order to compare and track yeast cell wall compositional changes during the β-glucan extraction process, ATR IR absorption spectra were recorded after 4 crucial extraction steps. Due to spectra similarities, only ATR IR absorption spectra of autolyzed cell walls (Figure 2) and β-glucans after organic solvent treatment (Figure 3) are shown. ATR IR absorption spectra after autoclaving and ultrasonication are available as Supplementary Materials (Figures S1 and S2).

Broad region between 3600–2600 cm^−1^ can be assigned to O-H bond, N-H bond at 3500–3100 cm^−1^ and CH bond at 3000–2800 cm^−1^ [27,34,35,36]. Characteristic amide regions are found at 1720–1620 cm^−1^ (amide I), 1570–1470 cm^−1^ (amide II) and 1350–1250 cm^−1^ (amide III) [16,19,34,37]. Sugar compounds are represented by the 1200–900 cm^−1^ region [27,34,35,36].

Both spectra of autolyzed yeast cell walls and hot water-extracted β-glucans have characteristic β-glucan bands at 1065, 1038 and 890 cm^−1^. The anomeric region of carbohydrates at 890 cm^−1^ represents the β-1,3-linkage of β-glycosidic bond [31,34] and can also be attributed to C-H deformation [38].

Intensity of characteristic β-glucan bands at 1375, 1312, 1100, 1065, 1038 and 890 cm^−1^ [11,34,39] remain unchanged in all samples throughout the hot water extraction process. β-1,3/1,6-glucan characteristic band at 1160 cm^−1^ is not observed in our samples [34,36]. However, another band, which represents α-linked glucans is located at 1155 cm^−1^ [34,40]. The band at 1155 cm^−1^ is stronger after treatment with organic solvents, indicating the presence of low levels of α-glucan impurities in all autolyzed samples and hot water extracted. *Candida* species compared to *S. cerevisiae*, *S. pastorianus* and *P. pastoris* has stronger 1155 cm^−1^ band intensity, indicating higher α-glucan content in the sample. Other characteristic α-glucan bands are located at 1125 and 850 cm^−1^ [12,34,37,40,41,42]; however, no changes in band intensity are observed during extraction.

Bands from β-1,6-glucan and mannans, which appear at 920 and 911 cm^−1^, respectively, have a tendency to overlap [31,43] and, after autolysis, has a weak band at 917 cm^−1^. Intensity changes in shoulders at 1052 and 975 cm^−1^, as well as bands at 917 and 810 cm^−1^ is observed after autolysis and treatment with organic solvents. After treatment with organic solvents, a small shift from 917 cm^−1^ to 919 cm^−1^ is observed as well as lowered band intensity at 810 cm^−1^ in all samples. These changes could indicate higher mannans and mannoprotein content after autolysis compared to hot water-extracted samples. Autolyzed cell walls of *S. cerevisiae*, *S. pastorianus* and *P. pastoris* had slightly higher levels of mannans compared to *C. parapsilosis*, *C. guilliermondii*, *C. lusitaniae* both after autolysis and treatment with organic solvents. Another characteristic spectral band for β-1,6-glucan is located near 1730 cm^−1^ [31], which is not observed in our samples. The band at 1743 cm^−1^ represents C=O stretching and generally represents lipids [17,44]. However, some researchers also associate it with chitin [37,45,46,47]. Band intensity at 1743 cm^−1^ lowers after autolysis, indicating lower residual lipid content after extraction process. After treatment with organic solvents, the highest intensity of this band is observed in samples from *P. pastoris* and lowest in samples from *C. lusitaniae* and *S. pastorianus.*

Broad band at 1643 cm^−1^ is attributed to amide I and to the stretching vibrations of C=O and C=C groups [12,27], indicating the presence of residual proteins. However, the presence of shoulder at 1650 cm^−1^ might suggest presence of other molecules. The broad band between 1500 cm^−1^ and 1600 cm^−1^ can be attributed to proteins as well [17,25,37]. The small shoulder located at 1650 cm^−1^ has lower intensity after treatment with organic solvents in all samples compared to autolysis. Vibration of proteins at 1587 cm^−1^ and 1624 cm^−1^ [17,48,49] indicate traces of proteins, as well as bands at 1650, 1517 and 1312 cm^−1^, which represent various protein secondary structures [33,50]. The band between 1600–1500 cm^−1^ in hot water-extracted β-glucans from *C. guilliermondii* and *C. lusitaniae* possess a peak at 1517 cm^−1^, which could possibly indicate higher protein content compared to β-glucans from other yeasts [25,33].

The chitin characteristic peak is indicated by a shoulder near 3100 cm^−1^, which is attributed to N-H stretching [36] and shoulder at 1025 cm^−1^, which correspond to β-1,4-glycosidic linkage [35,48]. Other chitin characteristic bands are located and are hidden by protein bands at 1538 cm^−1^ (NH), 1375 cm^−1^ (CH_3_) and 1312 cm^−1^ (CN) [40,51]. The shoulder near 3100 cm^−1^ is slightly more strongly pronounced in *C. guilliermondii*, *C. lusitaniae* and *S. pastorianus* after treatment with organic solvents. Other chitin bands overlap with glucan bands and lay in a broad region of O-H stretching vibration at 3400-3200 cm^−1^, which are generally attributed to polysaccharides [12,19,42,46,52].

In the *S. cerevisiae* sample, after autolysis, differences compared to other samples occurred at 1405, 1625, 1585, 1517 and 1590 cm^−1^, which could indicate other types of impurities, possibly occurred due to errors in processing.

### 2.4. Nuclear Magnetic Resonance Spectroscopy

**Figure 4:**
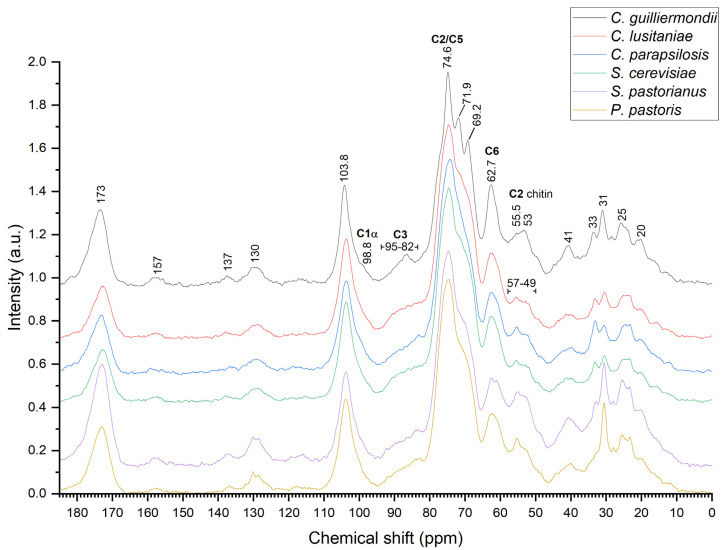
^13^C carbon molecule chemical shift of hot water-extracted β-glucans from 6 yeast strains: (from top to bottom) *Candida guilliermondii*, *C*. *lusitaniae*, *C*. *parapsilosis*, *Pichia pastoris*, *Saccharomyces cerevisiae* and *S*. *pastorianus*.

Solid-state NMR was used as the accompanying method to confirm the structure of isolated samples after the step of organic solvent treatment. The structure of β-1,3/1,6-glucans was confirmed by the presence of characteristic peaks at 104 ppm, 69 ppm and 62 ppm in NMR spectra [34,53]. Obtained data correspond with the data found in literature. Low levels of α-glucan are present in all samples due to weak signal of α-linked anomeric carbon found in the 99–102 ppm region (Figure 4) [12,34].

NMR also confirms the possible presence of molecules linked by β-1,4-glycosidic bond [54]. However, the region of 82–90 ppm is referred to several origins, such as C4 signal from β-1,4-glucan and chitin as well as C3 of α-1,3-glucan and β-1,3-glucan [34,55]. C4 linkage from mannose has a chemical shift around 83–80 ppm, which seems to be present in all samples [1,56]. The presence of chitin and protein characteristic bands confirmed by NMR indicate the presence of these molecules. *Pichia pastoris* possibly has higher chitin content compared to other yeasts due to stronger bands at 130, 55.5 (C2 carbon from chitin) and 25 ppm. β-glucans from *S. pastorianus* and *P. pastoris* might possess more chitin than other yeasts. Also, stronger band intensity at 130, 31 and 25 ppm in *C. guilliermondii*, *S. cerevisiae* and *P. pastoris* indicate higher protein levels. Multiple peaks in the region of 160–110 ppm belongs to aromatic amino acids and in the region of 50–10 ppm—to aliphatic amino acids [34]. The band at 157 ppm indicate a slightly higher amount of aromatic amino acids and at 31 ppm—aliphatic amino acids in samples from *C. guilliermondii* and *S. pastorianus* and *P. pastoris*, which correlate with data obtained by FT-IR spectroscopy. Chitin and protein band overlapping affects the signal intensity at 173, 130 and 25 ppm, which are both related to protein and chitin, corresponding to carbonyl group (C=O) and C2 carbon to the methyl group (CH_3_), respectively, indicating presence of the 1,4-linked β-chitin in other hot water-extracted β-glucans as well [53,54,55,57,58,59]. Stronger intensity near the 130 ppm and 30–20 ppm region indicates higher protein content, which is in agreement with the finding that overlapping aliphatic carbon bands of proteins and chitin at 173 ppm results in signal strengthening in both regions. Another molecule, which might have an impact on signal intensity, comes from lipids, which also overlap with bands from other molecules near 33 ppm [55].

## 3. Discussion

After the hot water extraction procedure, high molecular weight, water insoluble β-glucans with a typical appearance of β-1,3/1,6-glucan [12,19,25,31,34] were extracted from yeasts *C. guilliermondii*, *C*. *lusitaniae*, *C*. *parapsilosis*, *P. pastoris*, *S. cerevisiae* and *S*. *pastorianus*. The obtained data is in agreement with the data found in literature [12,27,40]. Molecules isolated by the hot water extraction method exhibit similar spectral features in all samples, however some differences such as presence of chitin, proteins and possibly other molecules are present. Based on ATR IR absorption spectral analysis, *C. guilliermondii*, *C. lusitaniae* has a slightly higher protein content compared to other yeasts. However, NMR analysis indicates the relatively higher protein and chitin content in *C. guilliermondii*, *S. pastorianus* and *P. pastoris.* In *P. pastoris*, the yeast cell wall structure as well as the β-glucan structure might be slightly different from other analyzed yeasts regarding higher chitin content or β-1,4-glucan presence in its structure [24]. Although we suggest that *P. pastoris* also exhibits β-1,3/1,6-glucan structure, compared to other yeasts, the band at 1027 cm^−1^ is stronger and could be attributed to β-1,4-glycosidic bond, which might indicate relatively higher chitin or β-1,4-glucan content in the cell wall of *P. pastoris* [12,33]. This could represent species-specific structural differences compared to other yeasts. Our findings indicate the presence of β-1,3-glucan with possible β-1,6-glucan side structure. Some differences in β-1,6-glucan content in the cell wall of analyzed *Candida* and *Saccharomyces* yeasts [53] are observed. Generally, these differences occur due to the isolation, drying methods or state of analyzed samples, aqueous or solid, as well as their source. As in this experiment, the isolation method, state of the sample and drying method were identical for all the samples, the only difference was the source, which could represent the occurred differences.

SEM analysis revealed charge accumulation in dry β-glucans samples. Vibrational spectra are highly sensitive to conformation and charge distribution [39], which means distortion of the obtained spectra and sample state: “dry”–solid or “wet”–solution, will affect the differences in IR spectra.

Overlapping of ATR IR absorption bands originating from different parts of complex molecules presents difficulties while interpreting the obtained spectra [30]. For example, we found that autolyzed cell walls of *Candida* species have stronger α-glucan band at 1155 cm^−1^ compared to *S. cerevisiae*, *S. pastorianus* and *P. pastoris*, which could be due to species-related structural cell wall differences. However, spectral band at 1155 cm^−1^, which is attributed to α-glucans, is controversial and together with spectral band at 1065 cm^−1^ might also represent the linear β-1,3-glucan structure [35] or could be attributed to β-1,3/1,6-glucan. Our suggestion is also supported by the absence of changes in characteristic α-glucan bands at 1125, 1023, 930, 850 and 765 cm^−1^. NMR spectroscopy analysis supports our suggestions about the presence of low levels of α-glucan due to a weak signal in the 99–102 ppm region.

Another difficult region for interpretation is in lipid region, which is also attributed to C-H stretching modes at 3000–2840 cm^−1^. Asymmetric stretching vibrational modes of CH_2_ and CH_3_ groups assigned to spectral bands located at 2925 and 2953 cm^−1^, respectively, as well as symmetric ones located at 2853 and 2875 cm^−1^ respectively, could also be found in polysaccharides [49,60,61], indicating that the presence of lipids cannot be attributed to these spectral bands in β-glucan samples. Furthermore, based on data found in literature, pure glucose and purified β-1,3/1,6-glucans possess spectral bands, which are generally attributed to lipids both in NMR spectra and ATR IR absorption spectra, from which we can conclude, that lipid content identification from β-glucans spectra is impeded. Still, in our samples, some level of lipid residues might be present.

Hot water-extracted β-glucans should maintain the β-1,3-glucan backbone and β-1,6-glucan side chain structure. One of the features of native structure β-glucan is insolubility in water, which is due to the presence of chitin molecules in its structure [16]. Chitin has some characteristic bands in amide I, amide II and amide III regions, which can interfere with result interpretation due to overlapping with protein bands, which are generally found in these regions. The small band at 1650 cm^−1^ observed after autolysis could indicate chitin presence in all samples. Band, which represent proteins at 1643 cm^−1^ could be attributed to bands from glucose, as solid-state glucose samples have a strong absorption in region 3000–2900 cm^−1^ and 1645 cm^−1^; however, these bands are not observed in the aqueous samples [61]. The weak water band at 1634 cm^−1^ can interfere with accurate analysis in this region, which confirms our hypothesis that the state of the molecule can impact the spectral changes and indicates that 1643 cm^−1^ could also have some contribution from β-glucan. Hence, some researchers associate this band (C=O) with chitin [45,46,47]. It could also be attributed to glucose tautomerism or cyclic glucan formation [62]. Β-glucan polymerization can also affect the stretching of the hydroxyl region at 1634 cm^−1^ [37,60]. Hence, it could impact recorded spectra in different ways, such as distortion, shifting, merging or intensity changes of the peaks found in these regions, which in turn alters the concentration calculations based on band and peak ratios, which might affect accuracy of the results and calculated concentration of molecules in the sample.

Difference in band pattern from autolyzed samples of *S. cerevisiae* indicate the presence of unidentified impurities. However, this difference occurred due to a human error during the cell wall preparation or washing stages and are not considered as significant because after treatment with organic solvents spectrum of *S. cerevisiae*, β-glucan does not differ significantly from other yeasts.

Our study confirms that analysis by FT-IR spectroscopy alone has difficulties when solid high molecular weight β-glucans are analyzed. Thus, the exact composition of the samples due to their relative impurity cannot be determined. Calculation of the polysaccharide, protein and lipid levels of the solid glucan samples from the band area (%) could be inaccurate due to overlapping regions, e.g., chitin band occurrence in protein regions or mannans overlapping with bands attributed to β-1,6-glucan. Overlapping could affect the intensity of the bands depending on the relative component concentration and impede the results. This feature might be useful to calculate the ratio of the bands in “wet” samples or β-glucans with lower molecular weight as well as soluble glucans. However, in order to preserve the native β-glucan structure, use of solid-state analysis is preferred. Thus, NMR spectroscopy together with FT-IR spectroscopy can improve structural analysis of insoluble high molecular weight β-glucans in solid state.

## 4. Materials and Methods

### 4.1. Yeast Growth

Six different yeast strains were kindly provided by the Microbiology and Biotechnology Department of Vilnius University LSC: *Candida guilliermondii*, *Candida lusitaniae*, *Candida parapsilosis*, *Pichia pastoris*, *Saccharomyces pastorianus* and *Saccharomyces cerevisiae*.

Yeasts were grown in 500-mL conical flasks with 250 mL of YPG broth in an orbital shaker for 48 h at 37 °C and 160 rpm, and afterward, biomass was collected by centrifugation at 5000× *g* and 4 °C, washed three times with distilled water and subjected to autolysis.

### 4.2. Extraction of Cell Wall Polysaccharides

In order to obtain crude, high molecular weight β-glucans and evaluate structural differences from different yeast sources, isolation was carried out by modified hot water extraction method originally described by Liu et al. [2] without protease treatment and without additional purification. Essential modification was that homogenization of autoclaved cell wall preparations was carried out by ultrasonication. Parameters for sonication were determined experimentally, using 40% of maximum output at 20 kHz frequency and power of 240 W (Sonics Vibra-Cell™, Newtown, CT, USA). Disruption consisted of 30:10 s (on: off) cycles for 15 min [23,24,25]. After sonication, supernatant and cell wall preparations were centrifuged and washed three times with distilled water 5000× *g* for 5 min at 4 °C. Extracts after four critical steps, autolysis, autoclaving, ultrasonication and treatment with organic solvents, were dried and weighed in order to calculate percent of β-glucan yield relative to initial yeast biomass and mass of the cell walls.

### 4.3. Scanning Electron Microscopy

Grinded β-glucan samples were placed on carbon conductive tabs for scanning electron microscopy analysis by a Tescan Vega3 scanning electron microscope (TESCAN ORSAY HOLDING, a.s., Brno-Kohoutovice, Czech Republic). In order to reduce charge accumulation, the samples were coated with a 2-nm-thick gold layer by magnetron sputtering system.

### 4.4. Fourier-Transform Infrared Spectroscopy

Attenuated total reflection (ATR) IR absorption spectra were measured after 4 main hot water extraction steps, autolysis, autoclaving, sonication and treatment with isopropyl alcohol at room temperature by using FT-IR spectrometer Alpha (Bruker Optic GmbH, Ettlingen, Germany) with an ATR module with a single reflection diamond crystal attached and equipped with an internal deuterated triglycine sulfate (DTGS) detector. ATR IR absorption spectra were recorded in the 400–4000 cm^−1^ spectral region with 4 cm^−1^ spectral resolution. Sixty-four interferograms were averaged and Fourier transformed into spectra. 3-Term Blackman-Harris apodization function, Power Spectrum phase correction mode and zero filling factor of 2 were applied for Fourier transformation. Before the measurement of spectra of each sample, the ATR crystal was cleaned with distilled water and ethanol and the background spectrum of ambient air was measured. Spectra were linear baseline corrected and normalized, and the break between 2750–1750 cm^−1^ was introduced due to the absence of spectral information and analyzed using OriginPro^®^ 2021 software (OriginPro 2021b 9.8.5.204 (Learning Edition), OriginLab Corporation, Northampton, MA, USA).

### 4.5. ^13^C Solid-State Nuclear Magnetic Resonance

Samples obtained after final step of hot water extraction were air-dried and subjected to solid-state NMR spectra registration using a Bruker AVANCE III HD spectrometer (Bruker Biospin GMBH, Ettlingen, Germany). The experiments were performed in a 9.4 T magnetic field using an Ascend wide bore superconducting magnet. The Larmor frequency for ^1^H and ^13^C was 400.16 and 100.62 MHz, respectively, and chemical shifts were referenced to adamantane. Magic angle spinning (MAS) measurements were performed using a Bruker 4 mm H/X CP-MAS probe-head, which is capable of spinning the sample up to 15 kHz rate. Samples were spun at a magic angle at 10 kHz rate using a 4 mm zirconia rotor at 300 K temperature. Spectra were accumulated by 1024 scans with repetition delay of 3 s. A rectangular variable contact time pulse (1 ms) shape was used in CP MAS experiments in order to fulfill Hartmann-Hahn matching conditions; for 1 H, the 90° pulse length was 3 μs. NMR spectra were processed using Topsin 3.2 software. Obtained data was transferred and analyzed with prior linear baseline correction and normalization using OriginPro^®^ 2021 software.

## 5. Conclusions

Hot water-extracted β-glucans from *S. cerevisiae*, *S. pastorianus*, *C. guilliermondii*, *C. lusitaniae*, *C. parapsilosis* and *P. pastoris* mostly resemble the structure of β-1,3-glucan with a small amount of β-1,6-glucan side chains. Protein impurities might be attributed to mannoprotein complexes still connected to β-1,3-glucan backbone. Low levels of chitin, mannan and α-glucan are present in all extracted samples. The β-glucan amount in the cell wall is species-related and depends on various environmental factors. However, strain-specific structure or content of β-glucans are not observed in ATR-IR absorption or NMR spectra. As for existing differences, they could have occurred due to natural differences of microorganisms, which are insignificant to a provide detailed conclusion. Stronger intensity of protein bands in ATR-IR absorption spectra, which in turn means higher protein levels connected to β-glucans, was established in *C. guilliermondii*, *P. pastoris* and *S. pastorianus*. Whether it is a structural peculiarity of the abovementioned yeasts or it is an error during the extraction process needs to be confirmed by future studies.

Significant differences in molecular mass, branching and structure of β-glucans is due to the different sources, which affect their biological activity [28]. However, in yeasts, these differences seem insignificant. Nevertheless, they also might determine differences in the biological activity of obtained β-glucans. This opens possibilities for the fundamental research of immune response to various yeast-caused infections (blood, gut, urogenital, etc.) as well as immunostimulation possibilities by different yeast β-glucans. Differences in cell wall composition and structure of β-glucan preparations from different yeast sources could result in different immune response.

β-glucan methods of polysaccharide determination are time-consuming and do not reveal the molecular structure. Application of “dry” analytical methods, such as FT-IR and NMR spectroscopy, are less time-consuming as samples require little or no preparation and could give an advantage when analyzing complex molecules, such as β-glucans. However, negative effects, such as “noise” and broad overlapping bands, makes them less accurate when analyzing high molecular weight β-glucans isolated by hot water extraction. ATR-IR absorption and NMR spectra of β-glucans are strongly affected by state, purity, isolation and drying methods as well as molecular weight and branching of the analyzed molecules, which result in overlapping of bands originated from different molecules, band and peak shifting, as well as changes in signal intensity. Solid state FT-IR and NMR spectroscopy are great for the fast determination of native β-glucan structure. However, FT-IR spectroscopy is limited in defining the exact composition of the complex molecules, such as high molecular weight β-glucans in solid state. In order to obtain more details on β-glucan structure, additional methods, such as solid-state NMR spectroscopy, must be used together with FT-IR spectroscopy.

## Figures and Tables

**Table 1:** Percent (%) of dried cell wall extracts mass relative to yeast cell biomass during different stages of hot water extraction. Values are an average of triplicate observations; ±standard deviation.

Yeast	Autolysis	Autoclaving	Ultrasonication	Organic Solvent	β-Glucan Conc. Relative to Cell Wall Mass
*C. guilliermondii*	13.34 ± 2.06	8.30 ± 1.96	6.70 ± 0.94	5.26 ± 0.52	40.22 ± 2.42
*C. lusitaniae*	14.91 ± 2.09	9.13 ± 0.84	7.20 ± 0.82	5.54 ± 0.73	37.26 ± 1.99
*C. parapsilosis*	11.39 ± 0.98	7.68 ± 0.99	6.64 ± 0.66	5.69 ± 0.61	49.92 ± 1.41
*P. pastoris*	13.05 ± 0.97	7.90 ± 0.55	6.07 ± 0.21	4.99 ± 0.45	38.49 ± 1.70
*S. cerevisiae*	12.81 ± 0.76	9.71 ± 0.60	8.32 ± 0.70	6.87 ± 0.65	53.55 ± 2.52
*S. pastorianus*	15.41 ± 3.32	11.71 ± 2.43	10.23 ± 1.90	8.83 ± 1.63	57.78 ± 3.45

## Data Availability

Not applicable.

## Supplementary Materials

The following supporting information can be downloaded at: https://www.mdpi.com/article/10.3390/molecules27144616/s1, Figure S1: Linear baseline corrected and normalized ATR IR absorption spectra of β-glucans from different yeast strains after hot water treatment process: (from top to bottom) *C. guilliermondii, C. lusitaniae*, *C. parapsilosis*, *P. pastoris*, *S. cerevisiae*, *S. pastorianus*. Figure S2: Linear baseline corrected and normalized ATR IR absorption spectra of β-glucans from different yeast strains after sonication process: (from top to bottom) *C. guilliermondii*, *C. lusitaniae*, *C. parapsilosis*, *P. pastoris*, *S. cerevisiae*, *S. pastorianus*.

## Sample Availability

Samples of the compounds are available from the authors.
