# Supplementary material for: FT-IR Method Limitations for β-Glucan Analysis
Source: Molecules. 2022 Jul 20;27(14):4616. doi: 10.3390/molecules27144616 (PMC9318380; doi:10.3390/molecules27144616)
Supplement: Supplementary file 1 [file molecules-27-04616-s001.zip › molecules-1806645-supplementary materials.pdf]

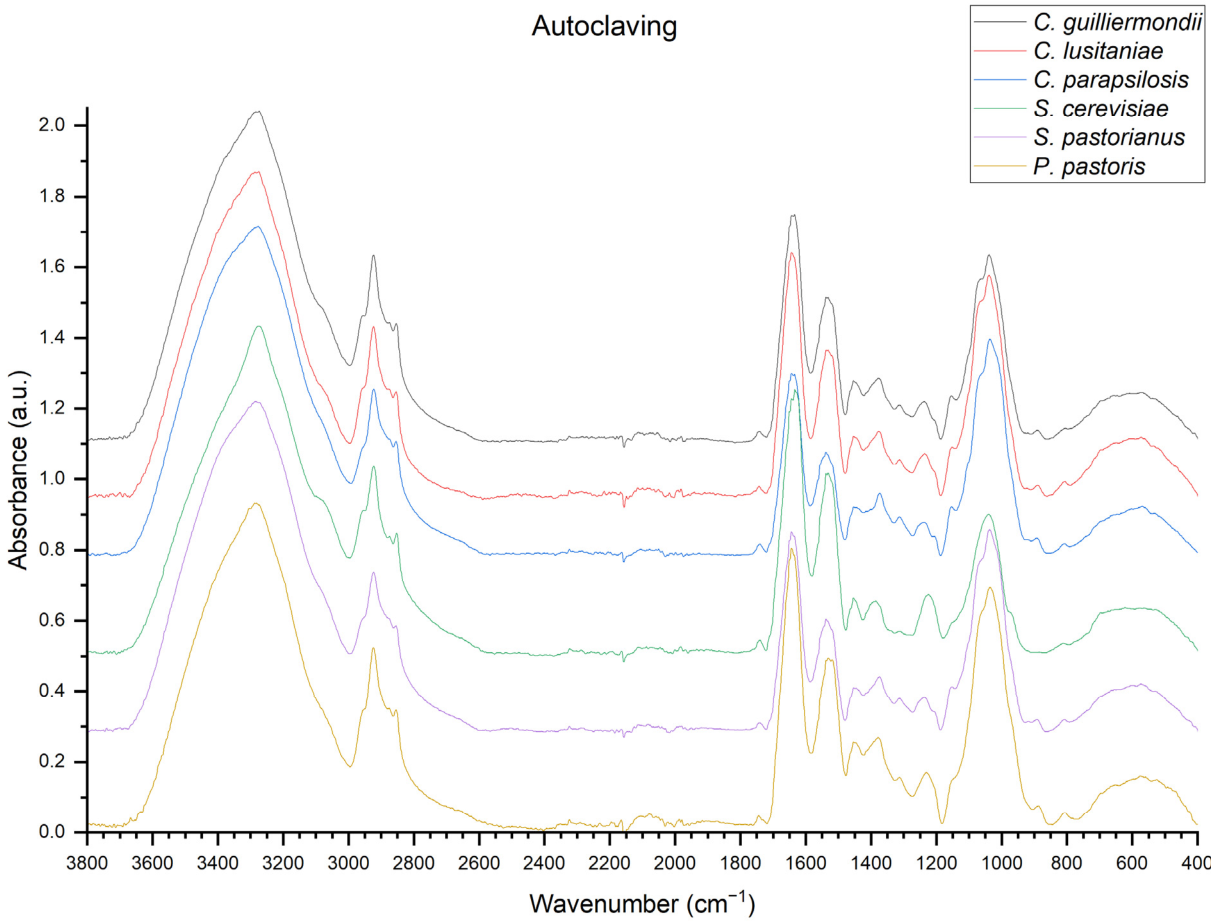

Figure S1. Linear baseline corrected and normalized ATR IR absorption spectra of β-glucans from different yeast strains after hot water treatment process: (from top to bottom) *C. guilliermondii, C. lusitaniae, C. parapsilosis, P. pastoris, S. cerevisiae, S. pastorianus*

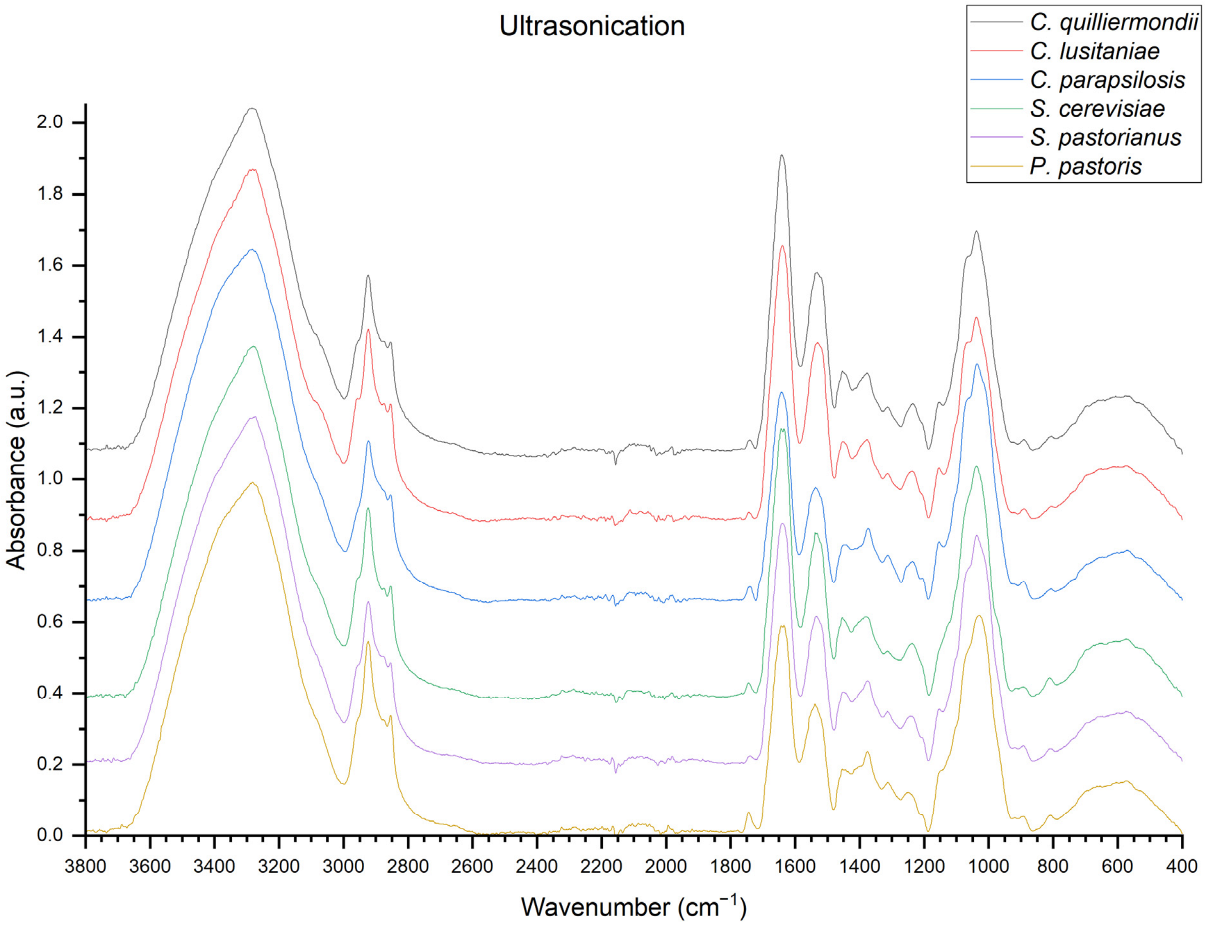

Figure S2. Linear baseline corrected and normalized ATR IR absorption spectra of β-glucans from different yeast strains after sonication process: (from top to bottom) *C. guilliermondii, C. lusitaniae, C. parapsilosis, P. pastoris, S. cerevisiae, S. pastorianus*
